# Factors associated with treatment outcome of acute post streptococcal glomerulonephritis among patients less than 18 years in Mekelle City, Public Hospitals, North Ethiopia

**DOI:** 10.1186/s13104-018-3794-7

**Published:** 2018-10-01

**Authors:** Lemlem Gebreselassie Gebreyesus, Atsede Fantahun Aregay, Kahsu Gebrekirstos Gebrekidan, Yisak Hagos Alemayehu

**Affiliations:** 1 Ayder Comprehensive Specialized Hospital, Mekelle University, Mekelle, Tigray Ethiopia; 2 School of Nursing, College of Health Science, Mekelle University, Mekelle, Tigray Ethiopia

**Keywords:** Acute post-streptococcal glomerular nephritis, Risk factors, Children

## Abstract

**Objective:**

To assess factors associated treatment outcomes of acute post streptococcal glomerular nephritis among patients less than 18 years old in Mekelle City Public Hospitals.

**Results:**

About 334 medical records c of children with acute post streptococcal glomerular nephritis were revised during the study period. Of these 244 (73.1%) had a positive outcome. acute post streptococcal glomerular nephritis was found to be statically significant associated with age < 5 years, duration of infection, the source of infection and length of stay in Hospital.

## Introduction

Acute post-streptococcal glomerulonephritis (APSGN) is an immune-mediated disease associated with acute respiratory tract infections and skin infections by β-hemolytic Streptococcus group A bacteria with clinical manifestation of edema, gross hematuria hypertension, proteinuria and oliguria persist more than 3 weeks [1]. Post-streptococcal glomerulonephritis (PSGN) is most common in children aged 5–12 years but not common before the age of 3 years. The most common acute glomerulonephritis is PSGN which is found mostly in developing countries and affecting more children than adults and leading to acute kidney injury, and potentially increasing morbidity in children and threatening life if delayed diagnosis and subsequently inaccurate treatment [2].

PSGN is reported from all over the world and the rates are higher in children than in adults. The incidence of PSGN in children was 24.3 cases per 100,000 people whereas in people older than 15 years, the estimated incidence is 2 cases per 100,000 people. Despite declining incidence of APSGN in many developed countries, there is still a significant burden in developing countries [3].

Regardless of the reduction in the worldwide incidence of PSGN, epidemics and clusters of cases of PSGN continue to appear. PSGN primarily affects children, aged 2–12 years in epidemic cases, with 10% in those below 2 years of age [4]. Prevalence of PSGN in the developing countries is still high, but its incidence declines in the industrial countries. Prognosis of APSGN is generally good; especially PSGN in children has a good outcome [5]. APSGN is principally diseases of poverty, because overcrowding and poor hygiene are prevailing which are the significant causes of morbidity and mortality in developing countries with low socioeconomic status [6].

The global prevalence of APSGN in is low particularly in industrialized nations because of easier and earlier access to competent medical treatment of streptococcal infections. The widespread use of fluorination of water was other factor because virulence factors in *Streptococcus pyogens* are reduced with fluoride exposure [7].

Most incidents of acute glomerulonephritis appear to be associated with a post-infectious state with known etiological agents [10]. It is known that the incidence of PSGN has recently decreased considerably in the developed world; the global burden of the disease has been less well quantified. Studies showed that PSGN incidence has fall insubstantially in Europe, South America & Asia. However, the disease may still cause substantial burden in indigenous communities and in poor, rural parts of developing countries such as Africa [8, 9].

Different studies reported PSGN patients developed different complications such as prolonged hypertension pulmonary edema, renal failure and proteinuria later in the clinical course and it needs early detection, early treated and prevention of source of infection like a sore throat and skin infection [1]. Though post-streptococcal complications are known to be common among Ethiopian children, but little is known about the morbidity and mortality associated with APSGN. Therefore, the aim of the study was to assess factors associated treatment outcome and of acute post-streptococcal glomerulonephritis among children less than 18 years old.

## Main text

### Methods

Hospital based retrospective cross-sectional study was conducted in Mekelle city which is the capital city of Tigray National Regional state, Ethiopia. All children less than 18 years who were admitted with a diagnosis of APSGN in the pediatric ward who had complete medical record in Ayder comprehensive specialized hospital & Mekelle Hospital from September 2013–August 2015 were included in the study. The sample size was 334 which was calculated using single proportion formula. A de-identified data from patient’s charts was used. Client’s chart was selected using simple random sampling based on the sampling frame prepared from Medical record of patients from Ayder comprehensive specialized hospital and Mekelle hospital. Structured checklist developed from different literature was used to collect data. The tool was pre tested in 10% of similar population which was not included in the study. Correction was done to the wording and other issues. To minimize bias, the data collectors were not employee of the hospitals included in the study. The collected data was checked for completeness and consistency before data entry and analysis. The data was coded and entered to Epi-info then, exported to SPSS version 20 for analysis. Descriptive statistics was computed, bivariate and multivariable logistic regressions were calculated to analyze the relationship between the dependent and independent variables. Association between variables were checked by considering p-value of 0.05 or less as the cut-off point for bivariate & multivariate regressions. Tests and tables were used to present the result.

### Results

#### Socio-demographic characteristics

A total of 334 records of children diagnosed with APSGN was included in this study. Most affected age group was between 5 and 9 years which was one hundred forty-two (42.5%). The age range of the participant patients was between 2 and 17 years with mean age of 8.6 years more (60.5%) of the study participants were males. Two hundred fifty-eight (77.2%) patients were from rural areas. The infection occurred during the dry and rainy seasons (Table 1).

**Table 1:** Demographic characteristic and clinical presentation of children < 18 years with PSAGN at comprehensive Specialized Hospital and Mekelle Hospital Mekelle. 2013–2015 (n = 334)

Variables	Category	Frequency	%
Age in year	< 5 years	49	14.7
	5–9 years	142	42.5
	10–14 years	131	39.2
	15–18 years	12	3.6
Sex	Male	202	60.5
	Female	132	39.5
Residential area	Urban	76	22.8
	Rural	258	77.2
Season of infection	Dry	171	51.2
	Rainy	163	48.8
Reasons for presentation^a^	General body swelling	274	82
	Facial swelling	60	18
	Bloody urine	180	53.9
	Oliguria	114	34.1
Nutritional status	Well nourished	52	15.6
	Under nourished	282	84.4
Duration of infection	Early (< 2 weeks)	58	17.4
	Late (> 2 weeks)	276	82.6
Source of infection	Sore throat	181	54.2
	Skin infection	110	32.9
	No history of infection	43	12.9
Length of hospitalization	< 2 weeks	76	22.8
	> 2 weeks	258	77.2
Complications^a^	Hypertension	214	64.1
	Encephalopathy	56	16.8
	Heart failure	78	23.4
	Pulmonary edema	60	18
	Renal failure	22	6.6

^a^More than one answer was possible

#### Clinical presentations

The most common chief complaints were body swelling 274 (82%) and hematuria 180 (53.9%). Two hundred eighty-two (84.4%) children were undernutrition according to the World Health Organization (WHO) parameter of nutritional status. About 258 patients came to Hospital later than 2 weeks of APSGN infection and 76 (22.8%) came as early as early 2 weeks and most of patients (77.2%) stayed in Hospital for longer than 2 weeks. The maximum length of stay in hospital was 62 days and minimum length of stay was 3 days. Hypertension was the most common complication which was developed by 214 (64.1%) patients (Table 1).

#### Laboratory indications

For all APSGN patient’s urinalysis was done for microscopic hematuria and protein urea. Both hematuria& protein urea were found in two 218 (65.3%), azotemia or nitrogen urea in blood found in 173 (51.8%) children, hyperkalemia and 158 (47.3%) children. ASO titer was done for 68 patients only and persistent microscopic hematuria could persevere up to 1–2 years after the initial presentation (Table 2).

**Table 2:** Laboratory Findings and management of Children With PSAGN Ayder compressive specialized Hospital and Mekelle Hospital Mekelle. 2013–2015 (n = 334)

Variables	Category	Frequency	%
Urine analysis	Hematuria	103	30.8
	Proteinuria	13	3.9
	Both	218	65.3
Hyperkalemia	Yes	158	47.3
	No	176	52.7
Anti-Streptococcal O (ASO) titer	Positive	41	12.3
	Negative	27	8.1
	Not done/not doc.	266	79.6
Critinin level (during discharge)	Normal < 1.2 mg/dl	273	81.7
	High ≥ 1.2 mg/dl	39	11.7
	Not done	22	6.6
Blood Urea Nitrogen (BUN) during discharge	Normal 7–20 mg/dl	265	79.3
	High > 20 mg/dl	46	13.8
	Not done	23	6.9
Dialysis	Yes	9	2.7
	No	325	97.3
Pharmacological management	Antibiotics	143	42.8
	Diuretics	332	99.4
	Anti hypertensive	334	100
Type of antihypertensive drugs	Nifidipine	178	53.3
	Enalaprile	34	10.2
	Others	122	36.5
Treatment outcome	Positive	244	73.1
	Negative	90	26.9

#### Pharmacological treatments

In this study, all patients were treated with either oral or parenteral furosemide. Two hundred and two (63.5%) patients took additional antihypertensive drugs such as Nifedipine, Hydralazine. Similarly, one hundred and forty-three (42.8) patients took antibiotics for the treatment of infection before presenting to hospital. In this study, the common anti-hypertensive drug next to furosemide was nifedipine which was taken by 178 (53.3%) patients (Table 2).

#### Treatment outcome of APSGN

Two hundred forty-four (73.1%) recovered immediately (positive outcome) and ninety (26.9) had negative outcome (20 died and 70 had follow-up) (Table 2).

#### Factors associated with treatment outcome of APSGN

In bivariate analysis age, place of residence, clinical future, nutritional status, duration of infection, source of infection and length of stay in hospital had association with outcome of acute post-streptococcal glomerulonephritis. Whereas, in multivariate logistic regression age, duration of infection, source of infection and length of stay were statically associated with acute post-streptococcal glomerulonephritis. Children less than 5 years old were four times at greater risk of APSGN than children older than 5 years [AOR = 4.593 with 95% CI (1.013–20.811)]. Children with duration of infection greater than 2 weeks were 4 times at greater risk than those who had duration of infection less than 2 weeks [AOR = 4.314 with 95% CI (1.101–15.491)], Children with evident source of infection had negative outcome as compared to those who had no evident source of infection [AOR = 3.909 with 95% CI (1.747–8.746)]. Length of stay in hospital was statistically associated with negative outcome of APSGEN, children who had hospital stay of greater than 2 weeks had negative out come as compared with those who had hospital stay of less than 2 weeks [AOR = 4.456 with 95% CI (2.426–8.184)] (Table 3).

**Table 3:** Factors associated with PSAGN at Ayder comprehensive specialized Hospital and Mekelle Hospital Mekelle. 2013–2015 (n = 334)

Variables	Outcome		COR 95% CI	AOR 95% CI
	Positive	Negative		
Age (years)				
< 5	39 (79.6%)	10 (20.4%)	3.900 (1.034–14.714)	4.593 (1.013–20.811)**
5–9	114 (79.2%)	30 (20.8%)	3.800 (1.143–12.628)	2.865 (0.735–11.178)
10–14	85 (67.9%)	44 (34.1)	1.932 (0.588–6.342)	1.510 (0.399–5.714)
15–18	6 (50%)	6 (50%)	1.00	1.00
Residential area				
Urban	63 (82.9%)	13 (17.1%)	1.00	1.00
Rural	181 (70.2%)	77 (29.8%)	0.485 (0.252–0.933)	0.580 (0.277–1.214)
Clinical				
General body swelling	191 (69.7%)	83 (30.3%)	0.304 (0.133–0.696)	0.480 (0.190–1.211)
Facial swelling	53 (88.3%)	7 (11.7%)	1.00	1.00
Nutritional status				
Well nutrition	31 (59.6%)	21 (40.4%)	1.00	1.00
Under nutrition	213 (75.5%)	69 (24.5%)	2.091 (1.128–3.875)	0.451 (0.116–1.750)
Duration of infection				
Less than 2 weeks	33 (56.9%)	25 (43.1%)	1.00	1.00
Greater than 2 weeks	211 (76.4%)	65 (23.6%)	2.459 (1.364–4.434)	4.314 (1.201–15.491)**
Source of infection				
Yes	226 (77.7%)	65 (22.3%)	4.829 (2.482–9.397)	3.909 (1.747–8.746)**
No	18 (41.9%)	25 (58.1%)	1.00	1.00
Length of stay in hospital (weeks)				
< 2	33 (43.4%)	43 (56.6%)	1.00	1.00
> 2	211 (81.8%)	47 (18.2%)	5.850 (3.365–10.168)	4.456 (2.426–8.184)**

** Highly significant in AOR and p-value < 0.05

### Discussion

Mortality and morbidity are the negative outcomes of APSGN which are known to be high in developing countries. In this study 244 (73.1%) patients had recovered from their illness immediately (positive outcome), whereus 90 (26.9%) had not recovered immediately (negative outcome). This was less as compared to the study conducted in Athens, Greece in which complete clinical and morphological recovery was observed in 92% children who had APSGN [10]. This finding was different as compared to a study done in Raze Ekman Hospital, Iran in which no mortality rate was reported during the study period [11, 12]. Whereas according to American Journal of nephron, in a Hospital in Turkey death was occurred in 20 to 25% of patients. Similarly, in a study conducted in Sudan Khartoum Hospital in 2012, 66% patients recovered from APSGN. The possible reasons for the difference in these findings might be because more complication was developed before presenting to hospital or health facility due to lack of infrastructure, not giving less priority to their disease and miss diagnosis of the disease.

Children who presented late with APSGN to hospital were 82.6% while only 17.4% children come early. In contrast to this finding, a study conducted in South Carolina found that, only 33% participants with PSGN [13] diagnosed lately. This might be due to the difference in socio economic status of the countries and difference in awareness of patients towards health care seeking behavior.

In this study, the time of presentation to hospital was statically significant with the outcome of APSGN [AOR = 4.314 (95% CI (1.201–15.491)]. Children present to hospital lately were four times at high risk to the negative outcome of APSGN. This is because late diagnose might lead to complication and severity which make them more exposed to mortality and morbidity. Delay in diagnosis or miss in diagnosing is common in children with PSGN, especially if visible hematuria is not a presenting complaint [13].

This study also found that, length of stay in hospital for greater than 2 weeks was statistically significant with negative outcome of APSGN [AOR = 4.456 (95% CI (2.426–8.184)]. As a result, children hospitalized for longer than 2 weeks f had four times higher risk of negative outcome than children who was admitted for less than 2 weeks. About 77.2% children admitted in hospital for > 2 weeks and the maximum length of stay in hospital was 62 days and the minimum stay was 3 days. This was in line with a study conducted in Malaysia which found that the shortest hospitalization was 4 days, while the longest was 66 days [14]. Another study conducted in Iran showed that the hospital stay of children with APSGN was range 2–26 days [12]. This variation might be due to difference in socioeconomic status, time gap and design of the study.

This study found that sore throat was the source of infection for 54.2% of children presented with APSGN and only 12% children had no past history (source) of infection. Whereas, a study conducted in Chile showed, skin infection was the likely source of 32 participants and only 5 participants had no past history (source) of infection [15].

In this study source of infection was 4 times high risk factors to negative outcomes of APSGN [AOR 3.909 with 95% CI (1.747–8.746)]. This was similar with a study done in Northern Territory, Australia that indicates social and environmental factors as risk factors of APSGN [16]. It is likely that overcrowding, lack of access to adequate water, heat, humidity, poor education and implementation of personal hygiene are all contributing factors to the risk of infection.

### Conclusion

In conclusion, From the total number of patients treated for post-streptococcal glomerulonephritis, 73.1% recovered immediately. The most common presentation/chief complaint was generalized body swelling. More than three-quarters of the patients have prolonged hospitalization for > 2 weeks and the common complication reported was Hypertension. The risk factors of APSGN in children was age, duration of infection source of infection and length of stay in the hospital. Since Ethiopia is one of the developing countries and there is high risk of skin and respiratory infections.

## Limitations

Some information about the clinical features and laboratory data in these patients was not available. Some patients discontinue long term follow-up and the end outcome was unknown.

## Abbreviations

AOR: Adjusted Odds Ratio
APSGN: acute poststreptococcal glomerulonephritis
ASO titer: anti streptococcal O titer
BUN: Blood Urea Nitrogen
PSGN: poststreptococcal glomerulonephritis
SPSS: Statistical Package for Social Sciences
WHO: World Health Organization
